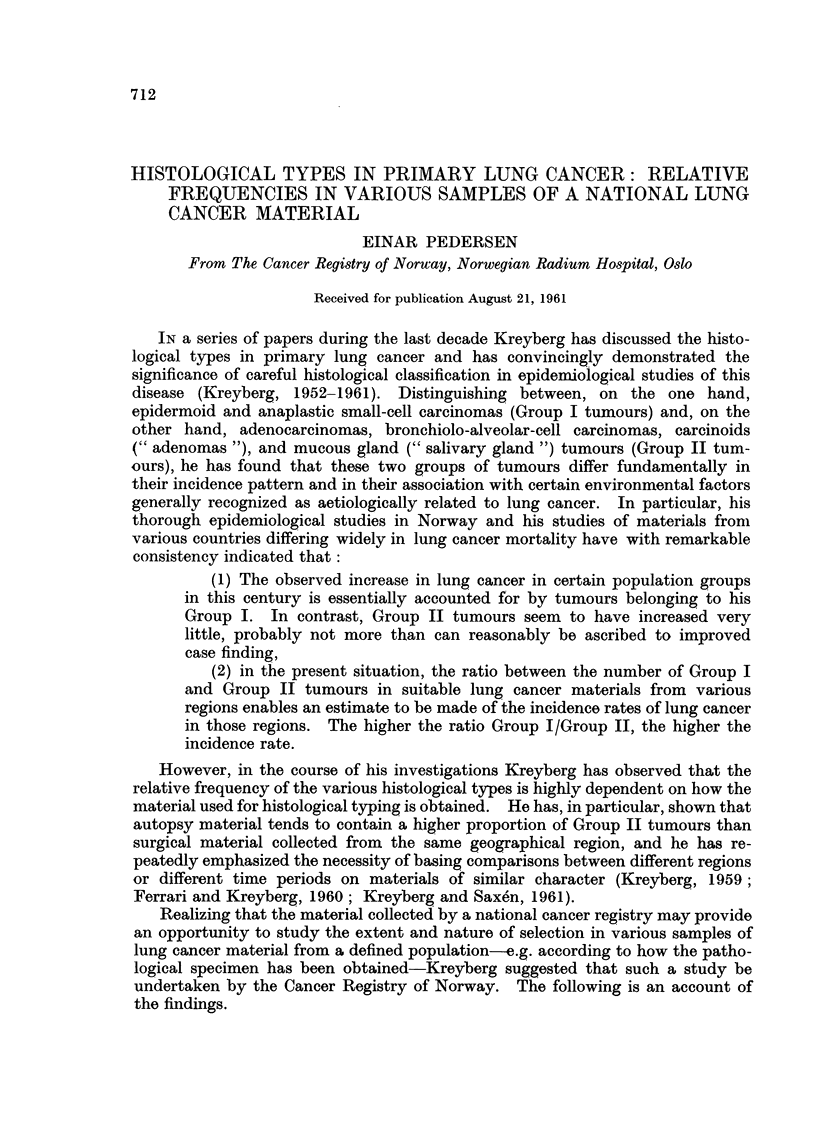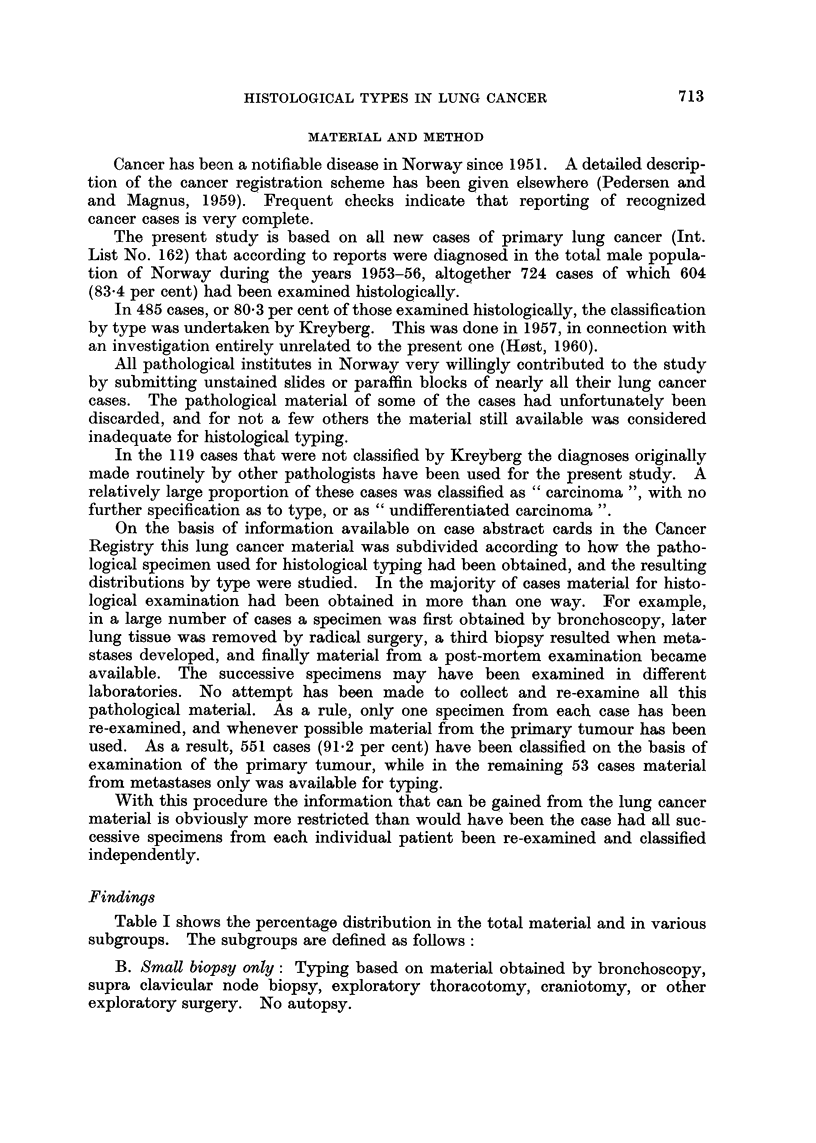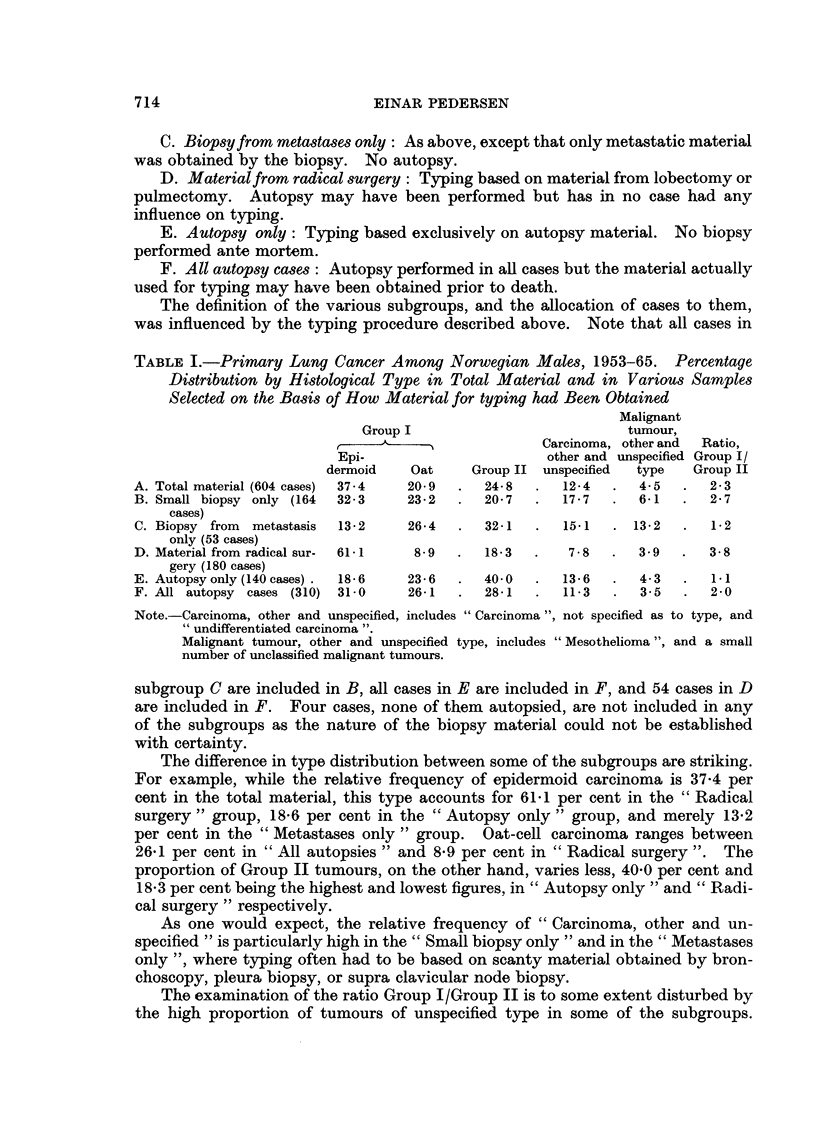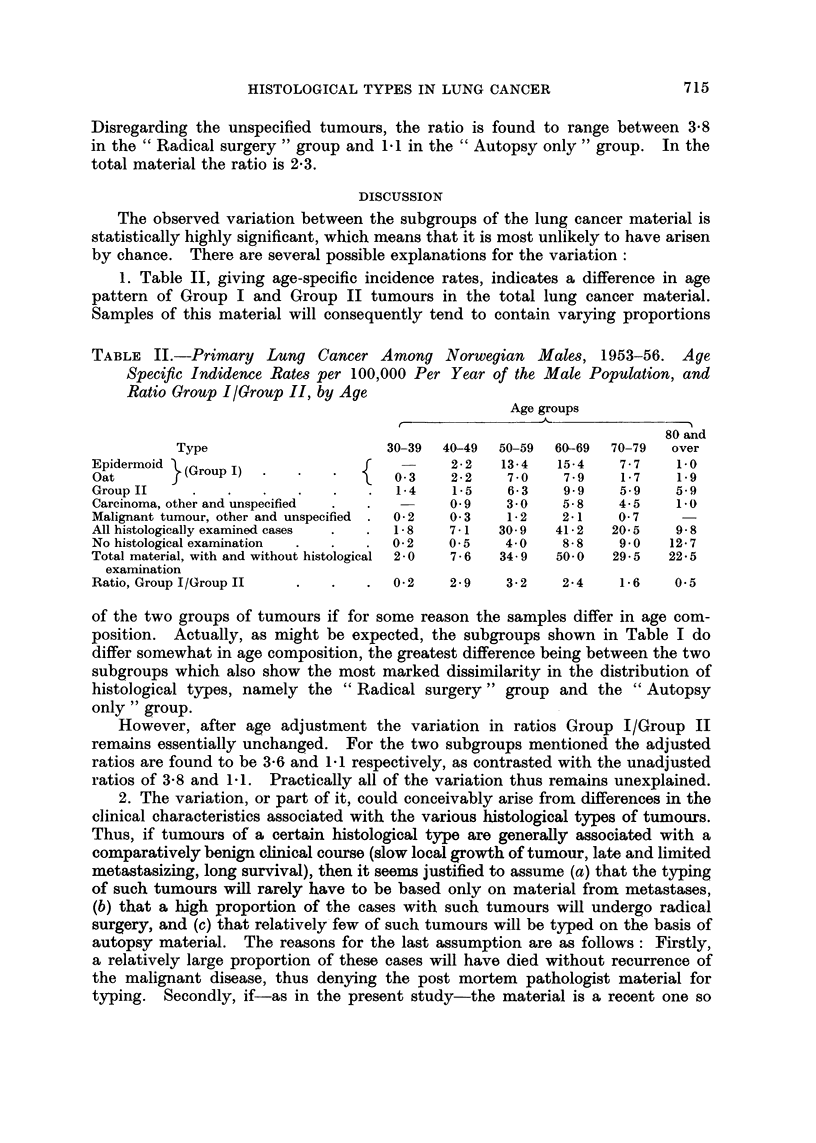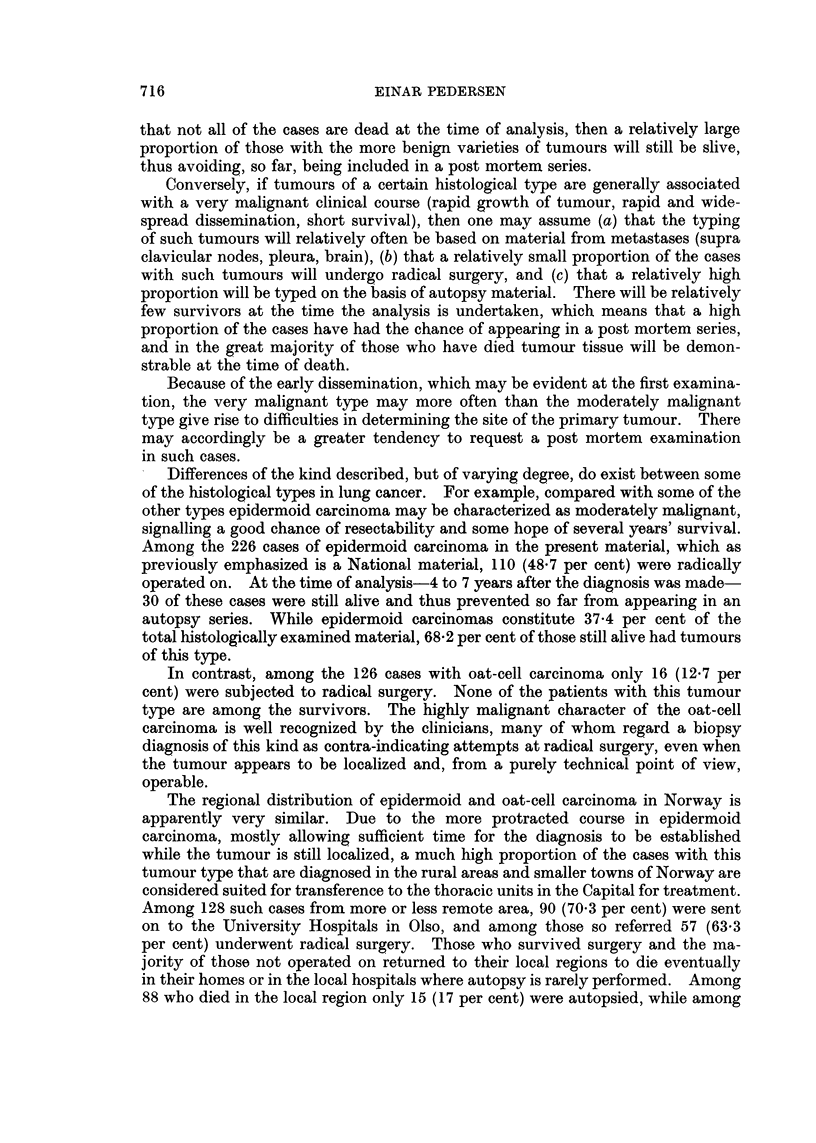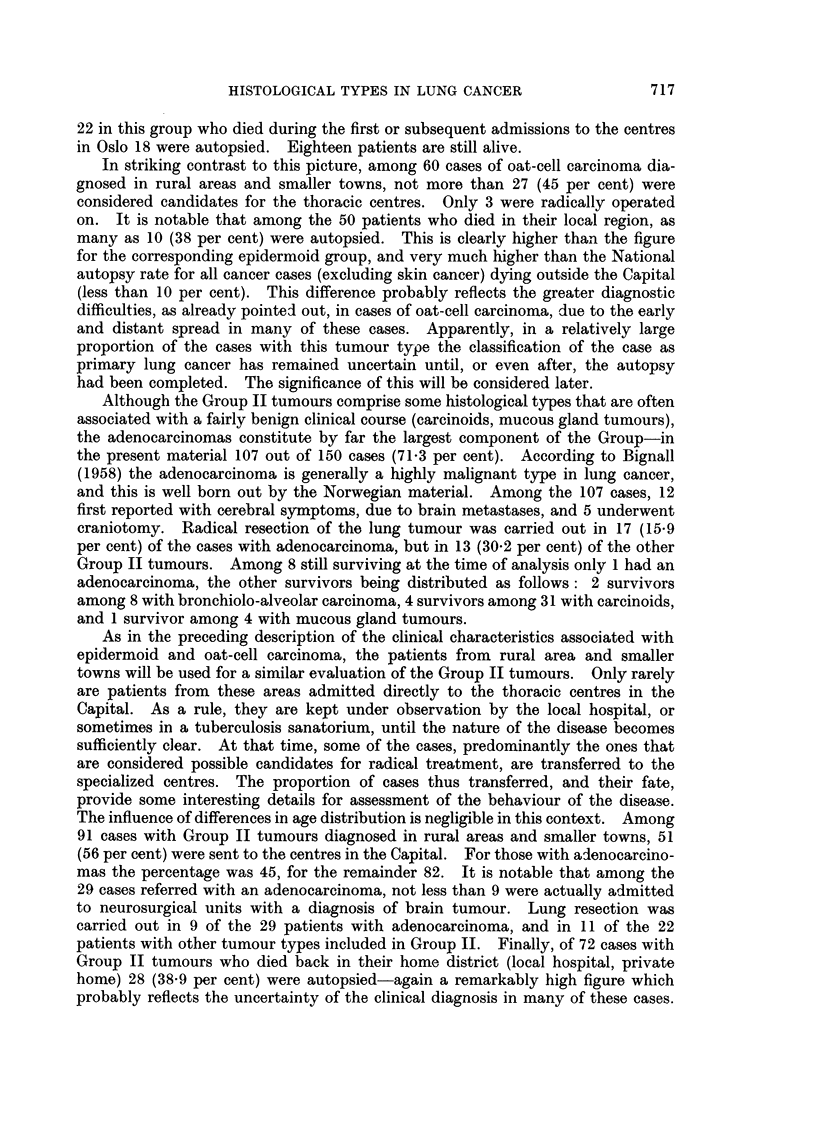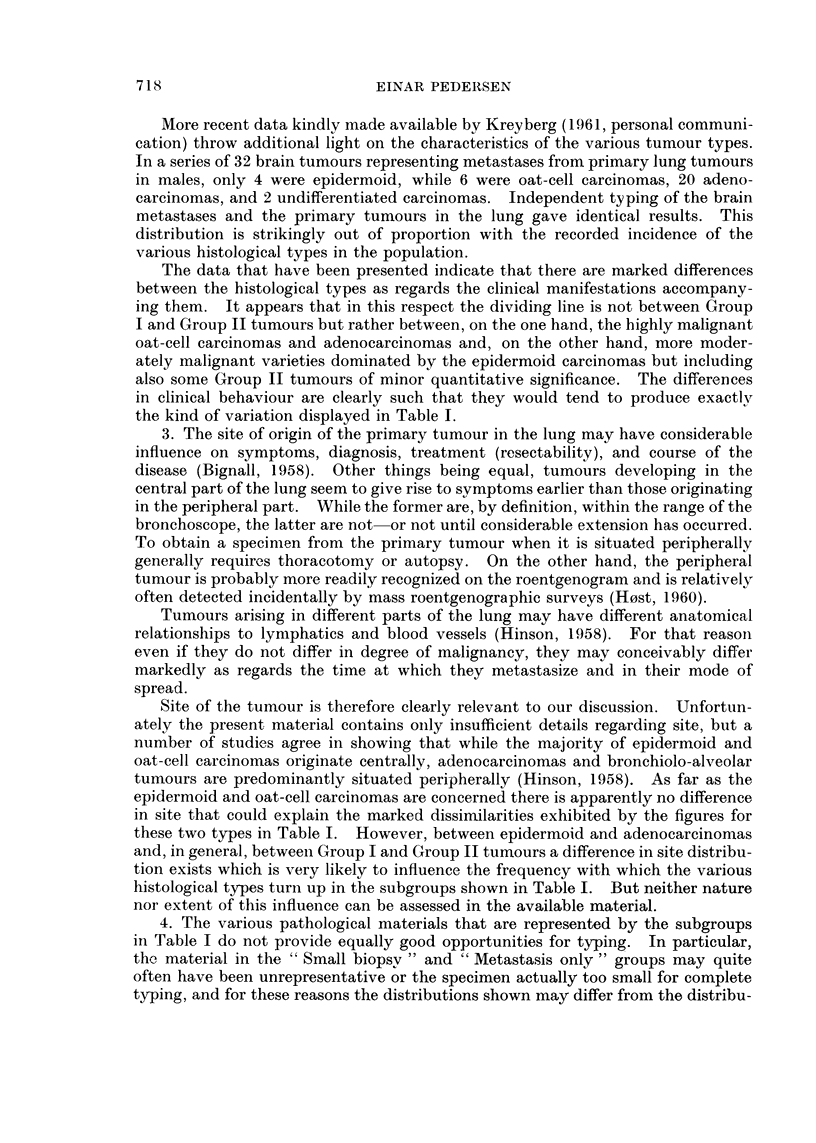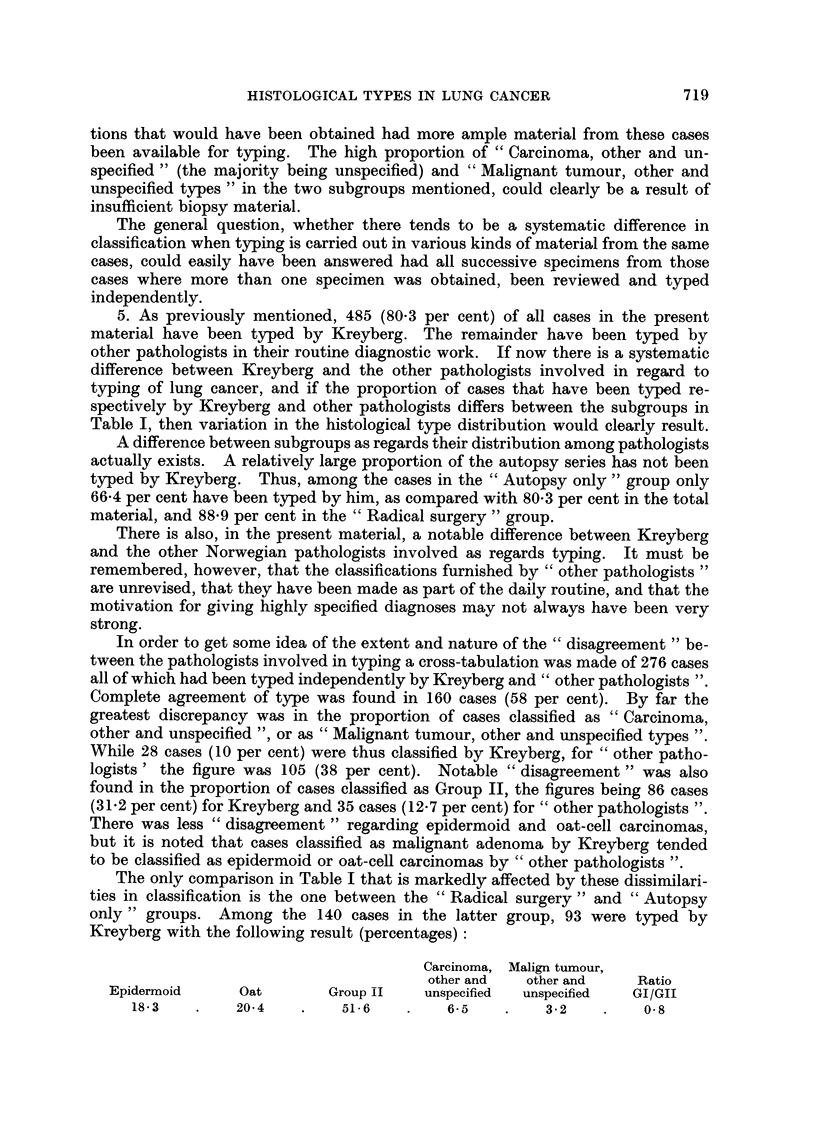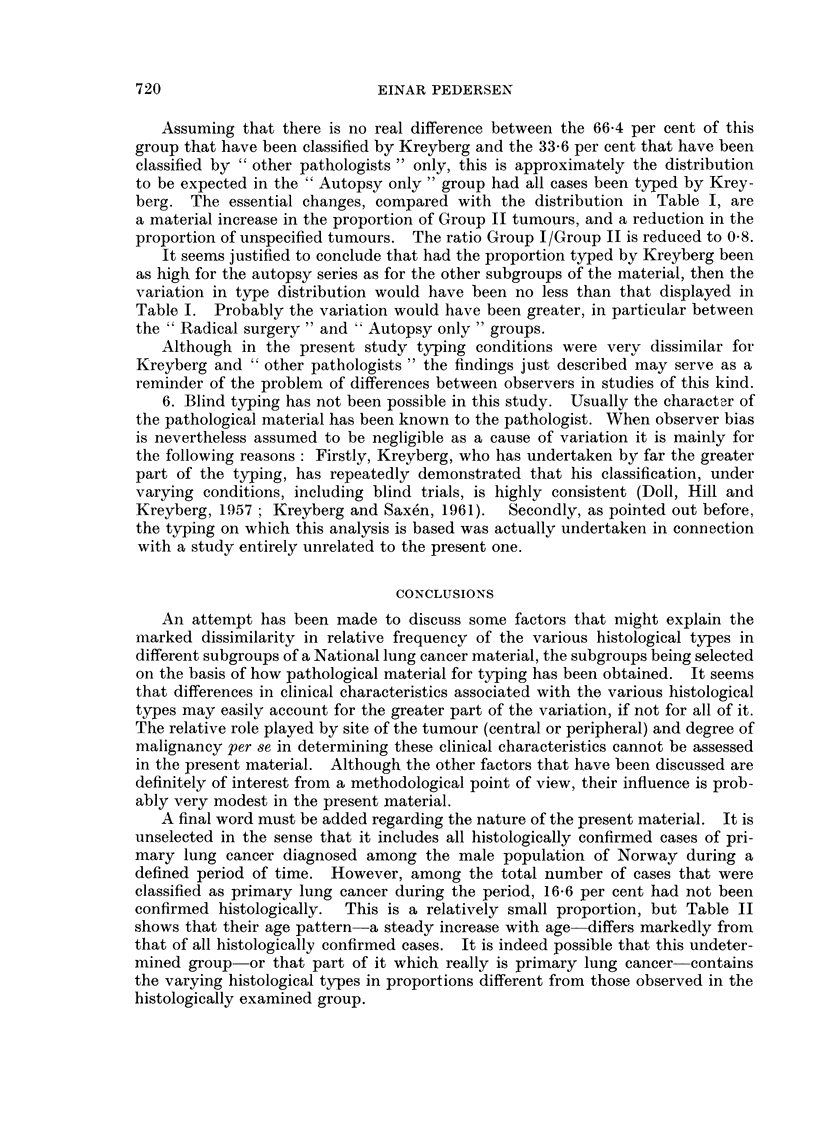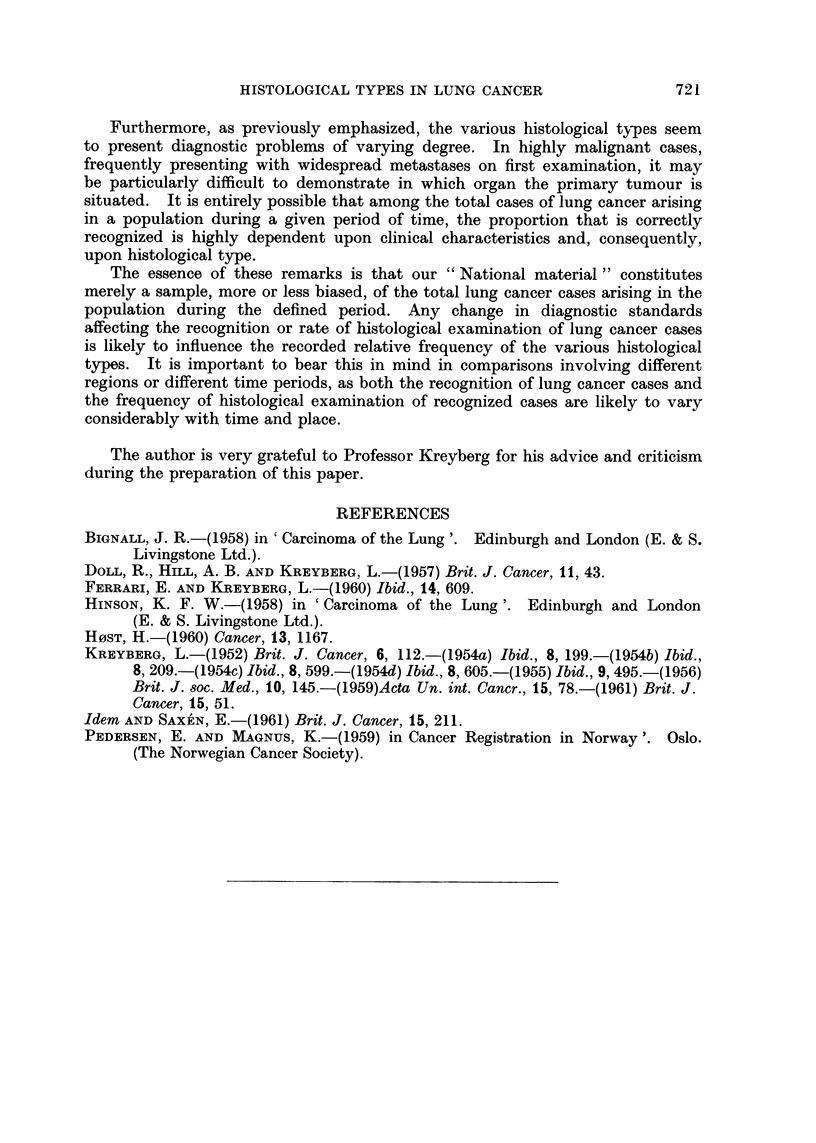# Histological Types in Primary Lung Cancer: Relative Frequencies in Various Samples of a National Lung Cancer Material

**DOI:** 10.1038/bjc.1961.81

**Published:** 1961-12

**Authors:** Einar Pedersen


### HISTOLOGICAL TYPES IN PRIMARY LUNG CANCER: RELATIVE FREQUENCIES IN VARIOUS SAMPLES OF A NATIONAL LUNG CANCER MATERIAL

EINAR PEDERSEN

*From The Cancer Registry of Norway, Norwegian Radium Hospital, Oslo*

Received for publication August 21, 1961

In a series of papers during the last decade Kreyberg has discussed the histological types in primary lung cancer and has convincingly demonstrated the significance of careful histological classification in epidemiological studies of this disease (Kreyberg, 1952–1961). Distinguishing between, on the one hand, epidermoid and anaplastic small-cell carcinomas (Group I tumours) and, on the other hand, adenocarcinomas, bronchiolo-alveolar-cell carcinomas, carcinoids (" adenomas "), and mucous gland (" salivary gland ") tumours (Group II tumours), he has found that these two groups of tumours differ fundamentally in their incidence pattern and in their association with certain environmental factors generally recognized as aetiologically related to lung cancer. In particular, his thorough epidemiological studies in Norway and his studies of materials from various countries differing widely in lung cancer mortality have with remarkable consistency indicated that:

(1) The observed increase in lung cancer in certain population groups in this century is essentially accounted for by tumours belonging to his Group I. In contrast, Group II tumours seem to have increased very little, probably not more than can reasonably be ascribed to improved case finding,

(2) in the present situation, the ratio between the number of Group I and Group II tumours in suitable lung cancer materials from various regions enables an estimate to be made of the incidence rates of lung cancer in those regions. The higher the ratio Group I/Group II, the higher the incidence rate.

However, in the course of his investigations Kreyberg has observed that the relative frequency of the various histological types is highly dependent on how the material used for histological typing is obtained. He has, in particular, shown that autopsy material tends to contain a higher proportion of Group II tumours than surgical material collected from the same geographical region, and he has repeatedly emphasized the necessity of basing comparisons between different regions or different time periods on materials of similar character (Kreyberg, 1959; Ferrari and Kreyberg, 1960; Kreyberg and Saxén, 1961).

Realizing that the material collected by a national cancer registry may provide an opportunity to study the extent and nature of selection in various samples of lung cancer material from a defined population—e.g. according to how the pathological specimen has been obtained—Kreyberg suggested that such a study be undertaken by the Cancer Registry of Norway. The following is an account of the findings.

### MATERIAL AND METHOD

Cancer has been a notifiable disease in Norway since 1951. A detailed description of the cancer registration scheme has been given elsewhere (Pedersen and and Magnus, 1959). Frequent checks indicate that reporting of recognized cancer cases is very complete.

The present study is based on all new cases of primary lung cancer (Int. List No. 162) that according to reports were diagnosed in the total male population of Norway during the years 1953–56, altogether 724 cases of which 604 (83·4 per cent) had been examined histologically.

In 485 cases, or 80·3 per cent of those examined histologically, the classification by type was undertaken by Kreyberg. This was done in 1957, in connection with an investigation entirely unrelated to the present one (Høst, 1960).

All pathological institutes in Norway very willingly contributed to the study by submitting unstained slides or paraffin blocks of nearly all their lung cancer cases. The pathological material of some of the cases had unfortunately been discarded, and for not a few others the material still available was considered inadequate for histological typing.

In the 119 cases that were not classified by Kreyberg the diagnoses originally made routinely by other pathologists have been used for the present study. A relatively large proportion of these cases was classified as " carcinoma ", with no further specification as to type, or as " undifferentiated carcinoma ".

On the basis of information available on case abstract cards in the Cancer Registry this lung cancer material was subdivided according to how the pathological specimen used for histological typing had been obtained, and the resulting distributions by type were studied. In the majority of cases material for histological examination had been obtained in more than one way. For example, in a large number of cases a specimen was first obtained by bronchoscopy, later lung tissue was removed by radical surgery, a third biopsy resulted when metastases developed, and finally material from a post-mortem examination became available. The successive specimens may have been examined in different laboratories. No attempt has been made to collect and re-examine all this pathological material. As a rule, only one specimen from each case has been re-examined, and whenever possible material from the primary tumour has been used. As a result, 551 cases (91·2 per cent) have been classified on the basis of examination of the primary tumour, while in the remaining 53 cases material from metastases only was available for typing.

With this procedure the information that can be gained from the lung cancer material is obviously more restricted than would have been the case had all successive specimens from each individual patient been re-examined and classified independently.

### *Findings*

Table I shows the percentage distribution in the total material and in various subgroups. The subgroups are defined as follows :

B. *Small biopsy only* : Typing based on material obtained by bronchoscopy, supra clavicular node biopsy, exploratory thoracotomy, craniotomy, or other exploratory surgery. No autopsy.

C. *Biopsy from metastases only* : As above, except that only metastatic material was obtained by the biopsy. No autopsy.

D. *Material from radical surgery* : Typing based on material from lobectomy or pulmectomy. Autopsy may have been performed but has in no case had any influence on typing.

E. *Autopsy only* : Typing based exclusively on autopsy material. No biopsy performed ante mortem.

F. *All autopsy cases* : Autopsy performed in all cases but the material actually used for typing may have been obtained prior to death.

The definition of the various subgroups, and the allocation of cases to them, was influenced by the typing procedure described above. Note that all cases in

TABLE I.—*Primary Lung Cancer Among Norwegian Males,* 1953–65. *Percentage Distribution by Histological Type in Total Material and in Various Samples Selected on the Basis of How Material for typing had Been Obtained*

| | Group I | | | | Malignant tumour, | |
|---|---|---|---|---|---|---|
| | Epidermoid | Oat | Group II | Carcinoma, other and unspecified | other and unspecified type | Ratio, Group I/ Group II |
| A. Total material (604 cases) | 37·4 | 20·9 | 24·8 | 12·4 | 4·5 | 2·3 |
| B. Small biopsy only (164 cases) | 32·3 | 23·2 | 20·7 | 17·7 | 6·1 | 2·7 |
| C. Biopsy from metastasis only (53 cases) | 13·2 | 26·4 | 32·1 | 15·1 | 13·2 | 1·2 |
| D. Material from radical surgery (180 cases) | 61·1 | 8·9 | 18·3 | 7·8 | 3·9 | 3·8 |
| E. Autopsy only (140 cases) | 18·6 | 23·6 | 40·0 | 13·6 | 4·3 | 1·1 |
| F. All autopsy cases (310) | 31·0 | 26·1 | 28·1 | 11·3 | 3·5 | 2·0 |

Note.—Carcinoma, other and unspecified, includes " Carcinoma ", not specified as to type, and " undifferentiated carcinoma ".
Malignant tumour, other and unspecified type, includes " Mesothelioma ", and a small number of unclassified malignant tumours.

subgroup *C* are included in *B*, all cases in *E* are included in *F*, and 54 cases in *D* are included in *F*. Four cases, none of them autopsied, are not included in any of the subgroups as the nature of the biopsy material could not be established with certainty.

The difference in type distribution between some of the subgroups are striking. For example, while the relative frequency of epidermoid carcinoma is 37·4 per cent in the total material, this type accounts for 61·1 per cent in the " Radical surgery " group, 18·6 per cent in the " Autopsy only " group, and merely 13·2 per cent in the " Metastases only " group. Oat-cell carcinoma ranges between 26·1 per cent in " All autopsies " and 8·9 per cent in " Radical surgery ". The proportion of Group II tumours, on the other hand, varies less, 40·0 per cent and 18·3 per cent being the highest and lowest figures, in " Autopsy only " and " Radical surgery " respectively.

As one would expect, the relative frequency of " Carcinoma, other and unspecified " is particularly high in the " Small biopsy only " and in the " Metastases only ", where typing often had to be based on scanty material obtained by bronchoscopy, pleura biopsy, or supra clavicular node biopsy.

The examination of the ratio Group I/Group II is to some extent disturbed by the high proportion of tumours of unspecified type in some of the subgroups.

Disregarding the unspecified tumours, the ratio is found to range between 3·8 in the " Radical surgery " group and 1·1 in the " Autopsy only " group. In the total material the ratio is 2·3.

### DISCUSSION

The observed variation between the subgroups of the lung cancer material is statistically highly significant, which means that it is most unlikely to have arisen by chance. There are several possible explanations for the variation :

1. Table II, giving age-specific incidence rates, indicates a difference in age pattern of Group I and Group II tumours in the total lung cancer material. Samples of this material will consequently tend to contain varying proportions

TABLE II.—*Primary Lung Cancer Among Norwegian Males,* 1953–56. *Age Specific Indidence Rates per* 100,000 *Per Year of the Male Population, and Ratio Group I/Group II, by Age*

| Type | Age groups 30–39 | 40–49 | 50–59 | 60–69 | 70–79 | 80 and over |
|---|---|---|---|---|---|---|
| Epidermoid (Group I) | — | 2·2 | 13·4 | 15·4 | 7·7 | 1·0 |
| Oat (Group I) | 0·3 | 2·2 | 7·0 | 7·9 | 1·7 | 1·9 |
| Group II | 1·4 | 1·5 | 6·3 | 9·9 | 5·9 | 5·9 |
| Carcinoma, other and unspecified | — | 0·9 | 3·0 | 5·8 | 4·5 | 1·0 |
| Malignant tumour, other and unspecified | 0·2 | 0·3 | 1·2 | 2·1 | 0·7 | — |
| All histologically examined cases | 1·8 | 7·1 | 30·9 | 41·2 | 20·5 | 9·8 |
| No histological examination | 0·2 | 0·5 | 4·0 | 8·8 | 9·0 | 12·7 |
| Total material, with and without histological examination | 2·0 | 7·6 | 34·9 | 50·0 | 29·5 | 22·5 |
| Ratio, Group I/Group II | 0·2 | 2·9 | 3·2 | 2·4 | 1·6 | 0·5 |

of the two groups of tumours if for some reason the samples differ in age composition. Actually, as might be expected, the subgroups shown in Table I do differ somewhat in age composition, the greatest difference being between the two subgroups which also show the most marked dissimilarity in the distribution of histological types, namely the " Radical surgery " group and the " Autopsy only " group.

However, after age adjustment the variation in ratios Group I/Group II remains essentially unchanged. For the two subgroups mentioned the adjusted ratios are found to be 3·6 and 1·1 respectively, as contrasted with the unadjusted ratios of 3·8 and 1·1. Practically all of the variation thus remains unexplained.

2. The variation, or part of it, could conceivably arise from differences in the clinical characteristics associated with the various histological types of tumours. Thus, if tumours of a certain histological type are generally associated with a comparatively benign clinical course (slow local growth of tumour, late and limited metastasizing, long survival), then it seems justified to assume (*a*) that the typing of such tumours will rarely have to be based only on material from metastases, (*b*) that a high proportion of the cases with such tumours will undergo radical surgery, and (*c*) that relatively few of such tumours will be typed on the basis of autopsy material. The reasons for the last assumption are as follows : Firstly, a relatively large proportion of these cases will have died without recurrence of the malignant disease, thus denying the post mortem pathologist material for typing. Secondly, if—as in the present study—the material is a recent one so that not all of the cases are dead at the time of analysis, then a relatively large proportion of those with the more benign varieties of tumours will still be slive, thus avoiding, so far, being included in a post mortem series.

Conversely, if tumours of a certain histological type are generally associated with a very malignant clinical course (rapid growth of tumour, rapid and widespread dissemination, short survival), then one may assume (*a*) that the typing of such tumours will relatively often be based on material from metastases (supra clavicular nodes, pleura, brain), (*b*) that a relatively small proportion of the cases with such tumours will undergo radical surgery, and (*c*) that a relatively high proportion will be typed on the basis of autopsy material. There will be relatively few survivors at the time the analysis is undertaken, which means that a high proportion of the cases have had the chance of appearing in a post mortem series, and in the great majority of those who have died tumour tissue will be demonstrable at the time of death.

Because of the early dissemination, which may be evident at the first examination, the very malignant type may more often than the moderately malignant type give rise to difficulties in determining the site of the primary tumour. There may accordingly be a greater tendency to request a post mortem examination in such cases.

Differences of the kind described, but of varying degree, do exist between some of the histological types in lung cancer. For example, compared with some of the other types epidermoid carcinoma may be characterized as moderately malignant, signalling a good chance of resectability and some hope of several years' survival. Among the 226 cases of epidermoid carcinoma in the present material, which as previously emphasized is a National material, 110 (48·7 per cent) were radically operated on. At the time of analysis—4 to 7 years after the diagnosis was made—30 of these cases were still alive and thus prevented so far from appearing in an autopsy series. While epidermoid carcinomas constitute 37·4 per cent of the total histologically examined material, 68·2 per cent of those still alive had tumours of this type.

In contrast, among the 126 cases with oat-cell carcinoma only 16 (12·7 per cent) were subjected to radical surgery. None of the patients with this tumour type are among the survivors. The highly malignant character of the oat-cell carcinoma is well recognized by the clinicians, many of whom regard a biopsy diagnosis of this kind as contra-indicating attempts at radical surgery, even when the tumour appears to be localized and, from a purely technical point of view, operable.

The regional distribution of epidermoid and oat-cell carcinoma in Norway is apparently very similar. Due to the more protracted course in epidermoid carcinoma, mostly allowing sufficient time for the diagnosis to be established while the tumour is still localized, a much high proportion of the cases with this tumour type that are diagnosed in the rural areas and smaller towns of Norway are considered suited for transference to the thoracic units in the Capital for treatment. Among 128 such cases from more or less remote area, 90 (70·3 per cent) were sent on to the University Hospitals in Olso, and among those so referred 57 (63·3 per cent) underwent radical surgery. Those who survived surgery and the majority of those not operated on returned to their local regions to die eventually in their homes or in the local hospitals where autopsy is rarely performed. Among 88 who died in the local region only 15 (17 per cent) were autopsied, while among

22 in this group who died during the first or subsequent admissions to the centres in Oslo 18 were autopsied. Eighteen patients are still alive.

In striking contrast to this picture, among 60 cases of oat-cell carcinoma diagnosed in rural areas and smaller towns, not more than 27 (45 per cent) were considered candidates for the thoracic centres. Only 3 were radically operated on. It is notable that among the 50 patients who died in their local region, as many as 10 (38 per cent) were autopsied. This is clearly higher than the figure for the corresponding epidermoid group, and very much higher than the National autopsy rate for all cancer cases (excluding skin cancer) dying outside the Capital (less than 10 per cent). This difference probably reflects the greater diagnostic difficulties, as already pointed out, in cases of oat-cell carcinoma, due to the early and distant spread in many of these cases. Apparently, in a relatively large proportion of the cases with this tumour type the classification of the case as primary lung cancer has remained uncertain until, or even after, the autopsy had been completed. The significance of this will be considered later.

Although the Group II tumours comprise some histological types that are often associated with a fairly benign clinical course (carcinoids, mucous gland tumours), the adenocarcinomas constitute by far the largest component of the Group—in the present material 107 out of 150 cases (71·3 per cent). According to Bignall (1958) the adenocarcinoma is generally a highly malignant type in lung cancer, and this is well born out by the Norwegian material. Among the 107 cases, 12 first reported with cerebral symptoms, due to brain metastases, and 5 underwent craniotomy. Radical resection of the lung tumour was carried out in 17 (15·9 per cent) of the cases with adenocarcinoma, but in 13 (30·2 per cent) of the other Group II tumours. Among 8 still surviving at the time of analysis only 1 had an adenocarcinoma, the other survivors being distributed as follows: 2 survivors among 8 with bronchiolo-alveolar carcinoma, 4 survivors among 31 with carcinoids, and 1 survivor among 4 with mucous gland tumours.

As in the preceding description of the clinical characteristics associated with epidermoid and oat-cell carcinoma, the patients from rural area and smaller towns will be used for a similar evaluation of the Group II tumours. Only rarely are patients from these areas admitted directly to the thoracic centres in the Capital. As a rule, they are kept under observation by the local hospital, or sometimes in a tuberculosis sanatorium, until the nature of the disease becomes sufficiently clear. At that time, some of the cases, predominantly the ones that are considered possible candidates for radical treatment, are transferred to the specialized centres. The proportion of cases thus transferred, and their fate, provide some interesting details for assessment of the behaviour of the disease. The influence of differences in age distribution is negligible in this context. Among 91 cases with Group II tumours diagnosed in rural areas and smaller towns, 51 (56 per cent) were sent to the centres in the Capital. For those with adenocarcinomas the percentage was 45, for the remainder 82. It is notable that among the 29 cases referred with an adenocarcinoma, not less than 9 were actually admitted to neurosurgical units with a diagnosis of brain tumour. Lung resection was carried out in 9 of the 29 patients with adenocarcinoma, and in 11 of the 22 patients with other tumour types included in Group II. Finally, of 72 cases with Group II tumours who died back in their home district (local hospital, private home) 28 (38·9 per cent) were autopsied—again a remarkably high figure which probably reflects the uncertainty of the clinical diagnosis in many of these cases.

More recent data kindly made available by Kreyberg (1961, personal communication) throw additional light on the characteristics of the various tumour types. In a series of 32 brain tumours representing metastases from primary lung tumours in males, only 4 were epidermoid, while 6 were oat-cell carcinomas, 20 adenocarcinomas, and 2 undifferentiated carcinomas. Independent typing of the brain metastases and the primary tumours in the lung gave identical results. This distribution is strikingly out of proportion with the recorded incidence of the various histological types in the population.

The data that have been presented indicate that there are marked differences between the histological types as regards the clinical manifestations accompanying them. It appears that in this respect the dividing line is not between Group I and Group II tumours but rather between, on the one hand, the highly malignant oat-cell carcinomas and adenocarcinomas and, on the other hand, more moderately malignant varieties dominated by the epidermoid carcinomas but including also some Group II tumours of minor quantitative significance. The differences in clinical behaviour are clearly such that they would tend to produce exactly the kind of variation displayed in Table I.

3. The site of origin of the primary tumour in the lung may have considerable influence on symptoms, diagnosis, treatment (resectability), and course of the disease (Bignall, 1958). Other things being equal, tumours developing in the central part of the lung seem to give rise to symptoms earlier than those originating in the peripheral part. While the former are, by definition, within the range of the bronchoscope, the latter are not—or not until considerable extension has occurred. To obtain a specimen from the primary tumour when it is situated peripherally generally requires thoracotomy or autopsy. On the other hand, the peripheral tumour is probably more readily recognized on the roentgenogram and is relatively often detected incidentally by mass roentgenographic surveys (Høst, 1960).

Tumours arising in different parts of the lung may have different anatomical relationships to lymphatics and blood vessels (Hinson, 1958). For that reason even if they do not differ in degree of malignancy, they may conceivably differ markedly as regards the time at which they metastasize and in their mode of spread.

Site of the tumour is therefore clearly relevant to our discussion. Unfortunately the present material contains only insufficient details regarding site, but a number of studies agree in showing that while the majority of epidermoid and oat-cell carcinomas originate centrally, adenocarcinomas and bronchiolo-alveolar tumours are predominantly situated peripherally (Hinson, 1958). As far as the epidermoid and oat-cell carcinomas are concerned there is apparently no difference in site that could explain the marked dissimilarities exhibited by the figures for these two types in Table I. However, between epidermoid and adenocarcinomas and, in general, between Group I and Group II tumours a difference in site distribution exists which is very likely to influence the frequency with which the various histological types turn up in the subgroups shown in Table I. But neither nature nor extent of this influence can be assessed in the available material.

4. The various pathological materials that are represented by the subgroups in Table I do not provide equally good opportunities for typing. In particular, the material in the " Small biopsy " and " Metastasis only " groups may quite often have been unrepresentative or the specimen actually too small for complete typing, and for these reasons the distributions shown may differ from the distributions that would have been obtained had more ample material from these cases been available for typing. The high proportion of " Carcinoma, other and unspecified " (the majority being unspecified) and " Malignant tumour, other and unspecified types " in the two subgroups mentioned, could clearly be a result of insufficient biopsy material.

The general question, whether there tends to be a systematic difference in classification when typing is carried out in various kinds of material from the same cases, could easily have been answered had all successive specimens from those cases where more than one specimen was obtained, been reviewed and typed independently.

5. As previously mentioned, 485 (80·3 per cent) of all cases in the present material have been typed by Kreyberg. The remainder have been typed by other pathologists in their routine diagnostic work. If now there is a systematic difference between Kreyberg and the other pathologists involved in regard to typing of lung cancer, and if the proportion of cases that have been typed respectively by Kreyberg and other pathologists differs between the subgroups in Table I, then variation in the histological type distribution would clearly result.

A difference between subgroups as regards their distribution among pathologists actually exists. A relatively large proportion of the autopsy series has not been typed by Kreyberg. Thus, among the cases in the " Autopsy only " group only 66·4 per cent have been typed by him, as compared with 80·3 per cent in the total material, and 88·9 per cent in the " Radical surgery " group.

There is also, in the present material, a notable difference between Kreyberg and the other Norwegian pathologists involved as regards typing. It must be remembered, however, that the classifications furnished by " other pathologists " are unrevised, that they have been made as part of the daily routine, and that the motivation for giving highly specified diagnoses may not always have been very strong.

In order to get some idea of the extent and nature of the " disagreement " between the pathologists involved in typing a cross-tabulation was made of 276 cases all of which had been typed independently by Kreyberg and " other pathologists ". Complete agreement of type was found in 160 cases (58 per cent). By far the greatest discrepancy was in the proportion of cases classified as " Carcinoma, other and unspecified ", or as " Malignant tumour, other and unspecified types ". While 28 cases (10 per cent) were thus classified by Kreyberg, for " other pathologists ' the figure was 105 (38 per cent). Notable " disagreement " was also found in the proportion of cases classified as Group II, the figures being 86 cases (31·2 per cent) for Kreyberg and 35 cases (12·7 per cent) for " other pathologists ". There was less " disagreement " regarding epidermoid and oat-cell carcinomas, but it is noted that cases classified as malignant adenoma by Kreyberg tended to be classified as epidermoid or oat-cell carcinomas by " other pathologists ".

The only comparison in Table I that is markedly affected by these dissimilarities in classification is the one between the " Radical surgery " and " Autopsy only " groups. Among the 140 cases in the latter group, 93 were typed by Kreyberg with the following result (percentages) :

| Epidermoid | Oat | Group II | Carcinoma, other and unspecified | Malign tumour, other and unspecified | Ratio GI/GII |
|---|---|---|---|---|---|
| 18·3 | 20·4 | 51·6 | 6·5 | 3·2 | 0·8 |

Assuming that there is no real difference between the 66·4 per cent of this group that have been classified by Kreyberg and the 33·6 per cent that have been classified by " other pathologists " only, this is approximately the distribution to be expected in the " Autopsy only " group had all cases been typed by Kreyberg. The essential changes, compared with the distribution in Table I, are a material increase in the proportion of Group II tumours, and a reduction in the proportion of unspecified tumours. The ratio Group I/Group II is reduced to 0·8.

It seems justified to conclude that had the proportion typed by Kreyberg been as high for the autopsy series as for the other subgroups of the material, then the variation in type distribution would have been no less than that displayed in Table I. Probably the variation would have been greater, in particular between the " Radical surgery " and " Autopsy only " groups.

Although in the present study typing conditions were very dissimilar for Kreyberg and " other pathologists " the findings just described may serve as a reminder of the problem of differences between observers in studies of this kind.

6. Blind typing has not been possible in this study. Usually the character of the pathological material has been known to the pathologist. When observer bias is nevertheless assumed to be negligible as a cause of variation it is mainly for the following reasons : Firstly, Kreyberg, who has undertaken by far the greater part of the typing, has repeatedly demonstrated that his classification, under varying conditions, including blind trials, is highly consistent (Doll, Hill and Kreyberg, 1957 ; Kreyberg and Saxén, 1961). Secondly, as pointed out before, the typing on which this analysis is based was actually undertaken in connection with a study entirely unrelated to the present one.

### CONCLUSIONS

An attempt has been made to discuss some factors that might explain the marked dissimilarity in relative frequency of the various histological types in different subgroups of a National lung cancer material, the subgroups being selected on the basis of how pathological material for typing has been obtained. It seems that differences in clinical characteristics associated with the various histological types may easily account for the greater part of the variation, if not for all of it. The relative role played by site of the tumour (central or peripheral) and degree of malignancy *per se* in determining these clinical characteristics cannot be assessed in the present material. Although the other factors that have been discussed are definitely of interest from a methodological point of view, their influence is probably very modest in the present material.

A final word must be added regarding the nature of the present material. It is unselected in the sense that it includes all histologically confirmed cases of primary lung cancer diagnosed among the male population of Norway during a defined period of time. However, among the total number of cases that were classified as primary lung cancer during the period, 16·6 per cent had not been confirmed histologically. This is a relatively small proportion, but Table II shows that their age pattern—a steady increase with age—differs markedly from that of all histologically confirmed cases. It is indeed possible that this undetermined group—or that part of it which really is primary lung cancer—contains the varying histological types in proportions different from those observed in the histologically examined group.

Furthermore, as previously emphasized, the various histological types seem to present diagnostic problems of varying degree. In highly malignant cases, frequently presenting with widespread metastases on first examination, it may be particularly difficult to demonstrate in which organ the primary tumour is situated. It is entirely possible that among the total cases of lung cancer arising in a population during a given period of time, the proportion that is correctly recognized is highly dependent upon clinical characteristics and, consequently, upon histological type.

The essence of these remarks is that our " National material " constitutes merely a sample, more or less biased, of the total lung cancer cases arising in the population during the defined period. Any change in diagnostic standards affecting the recognition or rate of histological examination of lung cancer cases is likely to influence the recorded relative frequency of the various histological types. It is important to bear this in mind in comparisons involving different regions or different time periods, as both the recognition of lung cancer cases and the frequency of histological examination of recognized cases are likely to vary considerably with time and place.

The author is very grateful to Professor Kreyberg for his advice and criticism during the preparation of this paper.

### REFERENCES

Bignall, J. R.—(1958) in ' Carcinoma of the Lung '. Edinburgh and London (E. & S. Livingstone Ltd.).

Doll, R., Hill, A. B. and Kreyberg, L.—(1957) *Brit. J. Cancer*, **11**, 43.

Ferrari, E. and Kreyberg, L.—(1960) *Ibid.*, **14**, 609.

Hinson, K. F. W.—(1958) in ' Carcinoma of the Lung '. Edinburgh and London (E. & S. Livingstone Ltd.).

Høst, H.—(1960) *Cancer*, **13**, 1167.

Kreyberg, L.—(1952) *Brit. J. Cancer*, **6**, 112.—(1954*a*) *Ibid.*, **8**, 199.—(1954*b*) *Ibid.*, **8**, 209.—(1954*c*) *Ibid.*, **8**, 599.—(1954*d*) *Ibid.*, **8**, 605.—(1955) *Ibid.*, **9**, 495.—(1956) *Brit. J. soc. Med.*, **10**, 145.—(1959)*Acta Un. int. Cancr.*, **15**, 78.—(1961) *Brit. J. Cancer*, **15**, 51.

*Idem* and Saxén, E.—(1961) *Brit. J. Cancer*, **15**, 211.

Pedersen, E. and Magnus, K.—(1959) in Cancer Registration in Norway '. Oslo. (The Norwegian Cancer Society).